# S12 Optimising health enhancing physical activity by using a human rights-based approach

**DOI:** 10.1093/eurpub/ckae114.250

**Published:** 2024-09-26

**Authors:** Sven Messing

**Affiliations:** FAU Erlangen, Department of Sport Science and Sport, Erlangen, Germany, University of Limerick, Ireland

## Abstract

The World Health Organization calls to employ a human rights-based approach for the implementation of the Global Action Plan on Physical Activity. However, research on physical activity promotion does usually not adopt a human rights perspective, even though there are overlaps with established fields of research such as health equity. The symposium is based on theoretical considerations on the potential status of physical activity as a human right, and aims to discuss how a human rights-based approach could be utilized to further develop physical activity promotion for vulnerable target groups.

Presentation 1 describes a framework that supports the use of a human rights-based approach to promote physical activity for vulnerable groups. The framework is based on four criteria (availability, accessibility, acceptability, and quality) which are discussed in the other presentations of the symposium. Presentation 2 focuses on the rights of persons with disabilities and investigates the perceptions of disability rights groups on the #Wethe15 campaign after the 2021 Paralympic Games. Presentation 3 systematically analyses a physical activity program for women in difficult life situations using a human rights-based approach based on semi-structured interviews.

Interaction with the audience will be stimulated by an interactive discussion on how a human rights-based approach could be integrated into PA promotion research, and whether this would be an added value from a political and scientific perspective.

